# Spatial and Temporal Selectivity of Translational Glass Patterns Assessed With the Tilt After-Effect

**DOI:** 10.1177/20416695211017924

**Published:** 2021-05-21

**Authors:** Andrea Pavan, Adriano Contillo, Filippo Ghin, Rita Donato, Matthew J. Foxwell, Daniel W. Atkins, George Mather, Gianluca Campana

**Affiliations:** Department of Psychology, University of Bologna, Bologna, Italy; School of Psychology, University of Lincoln, Lincoln, UK; Elettra-Sincrotrone Trieste S.C.p.A., Trieste, Italy; Department of Child and Adolescent Psychiatry, Cognitive Neurophysiology, Faculty of Medicine of the TU Dresden, Dresden, Germany; Department of General Psychology, University of Padova, Padova, Italy; Human Inspired Technology Research Centre, University of Padova, Padova, Italy; Department of Psychology, University of York, York, UK; School of Psychology, University of Lincoln, Lincoln, UK; Department of General Psychology, University of Padova, Padova, Italy; Human Inspired Technology Research Centre, University of Padova, Padova, Italy

**Keywords:** dynamic translational Glass patterns, tilt after-effect, motion-form integration, selectivity to spatial frequency, selectivity to temporal frequency

## Abstract

Glass patterns (GPs) have been widely employed to investigate the mechanisms underlying processing of global form from locally oriented cues. The current study aimed to psychophysically investigate the level at which global orientation is extracted from translational GPs using the tilt after-effect (TAE) and manipulating the spatiotemporal properties of the adapting pattern. We adapted participants to translational GPs and tested with sinewave gratings. In Experiment 1, we investigated whether orientation-selective units are sensitive to the temporal frequency of the adapting GP. We used static and dynamic translational GPs, with dynamic GPs refreshed at different temporal frequencies. In Experiment 2, we investigated the spatial frequency selectivity of orientation-selective units by manipulating the spatial frequency content of the adapting GPs. The results showed that the TAE peaked at a temporal frequency of ∼30 Hz, suggesting that orientation-selective units responding to translational GPs are sensitive to high temporal frequencies. In addition, TAE from translational GPs peaked at lower spatial frequencies than the dipoles’ spatial constant. These effects are consistent with form-motion integration at low and intermediate levels of visual processing.

A critical problem in form vision concerns the neural mechanisms underlying the extraction of global form from local orientation cues encoded early in the visual system. Glass patterns (GPs; Glass, 1969) have been widely used to study the integration of local orientation signals into a percept of global form. GPs are composed by dot pairs (dipoles) whose orientations align to create a global form; by applying different geometric transformations, it is possible to change the spatial relationship between dipole orientations to create visual textures that convey specific global forms that are often translational/parallel, circular, radial, hyperbolic, or spiral patterns (Ohla et al., 2005; Seu & Ferrera, 2001; Wilson & Wilkinson, 1998; Wilson et al., 1997). GPs perception is characterized by two main processes: (a) local processing that is based on the detection of local dipoles’ orientation and (b) global processing that allows the observer to perceive the overall orientation of dipoles giving to the GP a specific global shape (Chen, 2009; Chung & Khuu, 2014; Pei et al., 2005; Wilson & Wilkinson, 1998).

Several psychophysical and cell recording studies investigated how the local pooling of orientation cues in GPs occurs to generate the perception of an overall coherent pattern (Dakin, 1997a; Dakin & Bex, 2001; Smith et al., 2002, 2007). The perception of the overall global form of GPs can change depending on the dipole orientation, and it is achieved by the pooling of local signal information extracted from the spatial relationship between the dots forming the dipoles. This relationship between local and global form gives versatility to the pattern, as various aspects of the local form can be changed while maintaining the overall global form, such as the spatial frequency content (Dakin & Bex, 2001), density (Dakin, 1997a), colour (Mandelli & Kiper, 2005), and contrast (Dakin & Bex, 2001; Lin et al., 2017; Wilson et al., 2004). This versatility allowed to investigate how the visual system pools local orientation signals into a global form percept (Chen, 2009; Dakin & Bex, 2001; Nankoo et al., 2012) and the neural substrates underlying the perception of different spatial configurations (Krekelberg et al., 2005; Makin et al., 2016; Ostwald et al., 2008; Rampone & Makin, 2020).

While different GP configurations have been used to study neural response in monkey early visual cortex (Smith et al., 2002, 2007), the stage at which translational GPs are processed in humans is still debated. Early research by Dakin (1997a) proposed the two-stage model that describes how the extraction of the local orientation cues take place and how the local cues are integrated to give rise to an overall global shape (Dakin, 1997a; Lin et al., 2017). According to this model, the local orientation of the dipoles that form a GP stimulates the receptive fields of neurons in different cortical columns. This causes an intercolumnar excitation that leads to the pooling of the local orientation signals in the second stage. The two-stage model has been supported by computational modelling and studies on the spatial filtering present in the visual system (Prazdny, 1986; Wilson & Wilkinson, 1998; Wilson et al., 1997; Zucker, 1986). While the two-stage model provides an outline for how a global pattern could be perceived, it does not identify the stage at which GP processing occurs. To this purpose, a recent neurostimulation study demonstrated that the discrimination of static and dynamic translational GPs could be impaired by modulating human early visual areas (V1/V2) activity via repetitive transcranial magnetic stimulation (rTMS; Pavan, Ghin, et al., 2017). This suggests not only the fundamental involvement of low-level visual areas but also that the temporary disruption of V1/V2 activity prevents the forwarding of visual information to higher-level visual areas.

To investigate the mechanisms underlying the perception of translational GPs, various manipulations can be applied. For instance, the perception of translational GPs has been investigated measuring the participants’ coherence discrimination thresholds (Nankoo et al., 2012, 2015). Nankoo et al. (2012) compared the participants’ discrimination thresholds of different kinds of static and dynamic GPs (e.g., vertical, horizontal, circular, radial, and spiral) and random dot kinematograms with dots drifting according to specific trajectories and inducing the perception of vertical/horizontal motion and complex motion (i.e., circular, radial, and spiral motion). Their study investigated whether coherence thresholds were more similar between dynamic and static GPs or between dynamic GPs and real motion with random dot kinematograms. Concerning translational GPs, the authors found that vertical and horizontal GPs of both types (i.e., dynamic and static) were the most difficult to detect compared to the complex configurations. This phenomenon is known as *the complexity advantage* (Lee & Lu, 2010). Another type of manipulation used to explore the processing beneath the perception of translational GPs is an adaptation paradigm called tilt after-effect (TAE). In this regard, Pavan et al. (2016) carried out a study in which they induced the TAE adapting to translational GPs. The TAE (Gibson & Radner, 1937) is a perceptual illusion in which a grating (or other oriented patterns) is perceived as oriented away from its actual orientation following prolonged exposure to an oriented stimulus (i.e., orientation adaptation). Pavan et al. (2016) found that adaptation to translational GPs produces a TAE similar to that reported in studies using oriented gratings (e.g., Clifford et al., 2000), though reduced in magnitude. The authors also found that TAE from translational GPs shows almost complete interocular transfer, suggesting that the effect is likely to rely on visual processing levels in which the global orientation of GPs is encoded by neurons that are mostly binocularly driven and orientation-selective. Based on the characteristics observed, Pavan et al. (2016) concluded that the neural populations responsible for GP perception are orientation-selective and encode the global orientation of the patterns. A robust TAE from adaptation to GPs indicates that the visual system performs an effective integration of the adapting pattern. The spatial and temporal properties of the adapting translational GP can be manipulated to modulate the strength of the resulting TAE and consequently infer the spatiotemporal selectivity of units involved in global form processing (e.g., see Ware & Mitchell, 1974 for spatial selectivity of the TAE).

In the present study, we manipulated the spatial and temporal properties of adapting translational GPs to induce the TAE. Two experiments were performed using the same adaptation paradigm as in Pavan et al. (2016). In Experiment 1, we aimed at investigating the temporal frequency selectivity of units responding to translational GPs. In particular, we manipulated the temporal frequency of the adapting translational GPs to assess the temporal selectivity of the TAE from translational (static and dynamic) GPs. In Experiment 2, we investigated the spatial frequency selectivity of orientation-selective neural populations responding to dynamic translational GPs by manipulating the dipoles’ interdot distance. We predict that if a stronger TAE is obtained when the adapting pattern has a low spatial frequency content (i.e., high interdot distance) and low temporal frequency, then this might suggest the involvement of orientation-selective units at low level of visual processing. In this case, we expect to obtain narrow tuning curves with a peak at low spatial and temporal frequencies, rather than broad tuning curves. In fact, previous studies showed that low-level visual areas such as V1/V2 are sensitive to lower spatial and temporal frequencies than high-level visual areas (De Valois et al., 1982; Foster et al., 1985; Henriksson et al., 2008).

## Experiment 1

The aim of Experiment 1 was to investigate the temporal selectivity of orientation-selective units responding to translational GPs. To this purpose, we adapted to static (0 Hz) and dynamic GPs and measured TAE with a test grating stimulus. The rationale was that if the neural populations responsible for the perception of oriented GPs are sensitive to the temporal frequency of the GP, then we would expect that the manipulation of the temporal frequency of the adapting pattern would modulate the magnitude of the induced TAE. In turn, this would suggest that dynamic translational GPs are encoded at a level in which the neurons extracting the global form from GPs are sensitive to the temporal content of the pattern (e.g., in V1/V2, V3A; Foster et al., 1985; Gaska et al., 1988; Mazer et al., 2002; Ostwald et al., 2008; Pavan et al., 2016; Priebe et al., 2006).

### Method

#### Participants

Four of the authors (A. P., F. G., M. J. F., and D. W. A.) and five naïve observers voluntarily participated in the experiment. All participants had normal or corrected to normal visual acuity, and viewing was binocular. Methods conformed to the World Medical Association Declaration of Helsinki (2013). This study was approved by the Ethics Committee of the University of Lincoln, and written informed consent was obtained from each participant before enrolment in the study.

#### Apparatus

The apparatus was the same as used in our previous study (Pavan et al., 2019). Stimuli were generated using MATLAB PsychToolbox (Brainard, 1997; Pelli, 1997) and displayed on a 20-inch HP p1230 monitor with a refresh rate of 85 Hz, with a screen resolution of 1,280 × 1,024 pixels. Each pixel subtended 0.032 deg (i.e., ∼1.9 arcmin). The mean luminance was 37.5 cd/m^2^, whereas the minimum and maximum luminance values were 0.08 cd/m^2^ and 74.6 cd/m^2^, respectively. The screen was gamma-corrected. Observers sat in a darkened room at 57 cm from the screen. The participant’s head was stabilized by using a chinrest.

#### Stimuli

**Figure 1.**
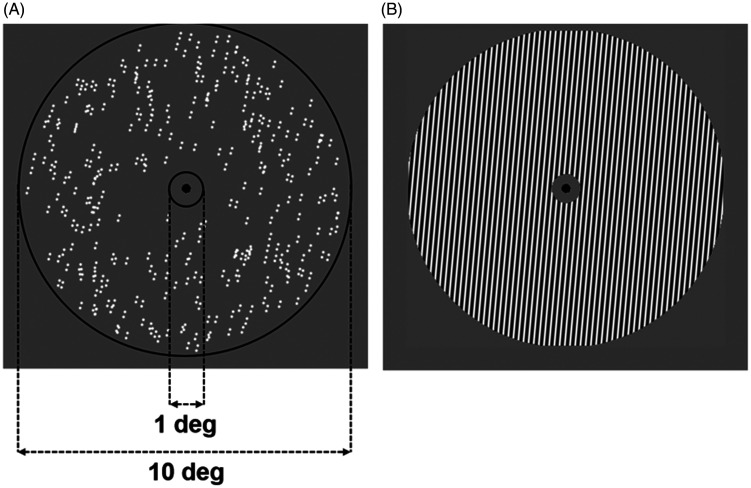
Representation of the stimuli used in Experiment 1. (A) Example of a translational GP oriented 15° clockwise from vertical (100% coherence). (B) Test grating used in Experiment 1 with a spatial frequency of 5.56 c/deg. For illustrative purposes, the grating is represented with higher contrast than the actual used in Experiment 1.

Adapting patterns were translational GPs consisting of 300 dipoles arranged in a circular annulus with an outer and inner radius of 5.0 deg and 0.5 deg, respectively (Figure 1A). Dots had a width of 0.1 deg and an interdot distance (centre-to-centre) of 0.18 deg (Clifford & Weston, 2005). Adapting GPs had a dipole density of 3.86 dipoles/deg^2^ and were always orientated 15° clockwise from vertical. GPs had 100% coherence. In Experiment 1, adapting GPs could be either stationary or dynamic. Dynamic GPs were created by displaying a sequence of independent frames. For each new frame (see Table 1 for the frame durations used), a new spatial arrangement of the dipoles was created, each with a total number of dipoles equal to 300, while their orientation remained constant (i.e., 15° clockwise from vertical). In dynamic GPs, the rapid succession of frames induces the perception of apparent motion along the orientation axis of the pattern even though there is no dipole-to-dipole correspondence between successive frames. Therefore, no coherent motion is present in this class of stimuli (Nankoo et al., 2012; Pavan, Bimson, et al., 2017; Ross, 2004; Ross et al., 2000).

**Table 1.** The First Row of the Table Reports the Adapting GPs Temporal Frequencies (Hz) Used in Experiment 1.

*Adapter temporal frequency (Hz)*						
1.32	2.65	5.30	10.59	21.19	42.37	84.75
*Frame duration (s)*						
0.7552	0.3776	0.1888	0.0944	0.0472	0.0236	0.0118

*Note*. The second row reports the frame duration (in seconds) used to obtain the corresponding temporal frequency. Given that the refresh rate of the screen was constant at 85 Hz, longer frame durations were obtained using multiples of a single frame duration (i.e., ∼0.0118 s).

The test pattern (Figure 1B) was a sinewave grating with a spatial frequency of 5.56 c/deg, corresponding to the inverse of the dipoles’ spatial constant (i.e., the interdot distance—see Figure 5 for more details). Therefore, the period of the test grating matched the dipoles’ interdot separation. The inner and outer radii of the test gratings were of the same size as those used for the GPs (i.e., 0.5 deg and 5 deg for inner and outer radii, respectively). The Weber contrast of each dot in the GP was 0.99 and the contrast of the test grating was 0.5 (Michelson contrast).

#### Procedure

Participants completed two main conditions: (a) a no-adaptation condition (baseline) and (b) an adapting condition, in which they were adapted to either static or dynamic translational GPs.

#### No-Adaptation Condition (Baseline)

In the baseline condition, participants reported whether the orientation of a grating (as described in the Stimuli section) was tilted either clockwise or counterclockwise from the vertical meridian, by pressing one of two designated keys on a standard English computer keyboard (“M” and “Z” for clockwise and counterclockwise responses, respectively; method of single stimuli [MSS]; Morgan et al., 2012). First, a fixation dot was presented in the centre of the screen for 1 second, followed by the test grating presented for approximately 0.035 seconds (i.e., 3 frames at 85 Hz). We used a very brief test grating for two reasons: (a) The TAE is stronger in magnitude with brief test durations (Wolfe, 1984) and (b) to maximize the effect of adaptation, that in the case of GPs fades away very rapidly (Pavan et al., 2016).

Two simple one-up/one-down staircases (Levitt, 1971) were used to estimate the point of subjective vertical (PSV) for each observer, that is, the orientation of the grating for which the observers were at chance in responding that the testing pattern was tilted either clockwise or counterclockwise from the vertical meridian. The two staircases were randomly interleaved. The first staircase started by displaying the grating oriented 10° clockwise from vertical, whereas the second staircase started by displaying the grating oriented 10° counterclockwise from vertical. On subsequent trials, the grating was rotated in the direction opposite to that reported by the observer (Pavan et al., 2016). The test grating was rotated by 1 deg until the first reversal and then by 0.5 deg. There was no visible cue or reference frame for vertical. Each staircase terminated after 75 trials. In general, observers performed two baseline blocks, with each block consisting of two interleaved one-up/one-down staircases (Levitt, 1971). Author “F. G.” performed three baseline blocks, whereas one naïve participant (“BH”) performed one baseline block.

The PSV was estimated from the staircase by fitting a cumulative Gaussian function to the binned data. The cumulative Gaussian function related the probability of clockwise responses “*p(clockwise)*” to the orientation of the grating (*θ*). The cumulative Gaussian had the following form:

(1) $$p\left(\mathrm{clockwise}\right)=\frac{1}{2}\left[\mathrm{erf}\left(\frac{\theta -m}{\sqrt{2\sigma^{2}}}\right)+1\right]$$

where *erf* is the error function

(2) $$\mathrm{erf}\left(x\right)=\frac{2}{\sqrt{\pi }}\int_{0}^{x}e^{-t^{2}}\text{d}t$$

*m* is the midpoint (i.e., the PSV) of the function and σ is standard deviation of the Gaussian function. The function in Equation 1 spans from *p* = 0 (for largely counterclockwise stimuli) to *p* = 1 (for largely clockwise stimuli). The slope *s* of the cumulative Gaussian around the midpoint, defined as the local variation of the cumulative Gaussian corresponding to an orientation variation of 1 deg, is

(3) $$s=\frac{\pi }{180\sqrt{2\pi \sigma^{2}}}$$

The PSV values estimated from each staircase of the baseline blocks (i.e., four staircases in total but six staircases for FG and two staircases for BH) were averaged, and the resulting value was considered as the subjective vertical. The mean value of the PSV for the no-adaptation baseline condition was obtained as a weighted average, using as weights the standard error (SE) values of the PSV obtained from the curve fitting routine.

#### Adaptation Condition

The task in the adaptation condition was the same as in the baseline condition. Observers were asked to judge the orientation of the grating after adaptation to either static or dynamic translational GPs. We used a top-up adaptation paradigm in which on the first trial observers were adapted for 30 seconds and then on each subsequent trial a top-up adaptation of 5 seconds was presented. Shortly after the adaptation period (i.e., 1 frame, ∼0.0118 seconds), the test grating was presented. The orientation of the test grating was varied by a simple one-up/one-down staircase (Levitt, 1971). The staircase could start presenting the test grating tilted 20 deg either clockwise or counterclockwise from vertical and used the same step sizes as in the baseline condition. Observers completed eight different adaptation conditions, in which the adapting temporal frequency was varied (Table 1). We also included a condition in which the adapting GP was static (0 Hz). The adapting orientation was kept constant within each staircase and was 15 deg clockwise from vertical. In the adaptation condition, the staircase terminated after 60 trials. It should be noted that for the adaptation conditions we used slightly less trials than in the no-adaptation conditions (i.e., 60 and 75 trials, respectively), this to limit fatigue and motivation drop in our participants.

**Figure 2.**
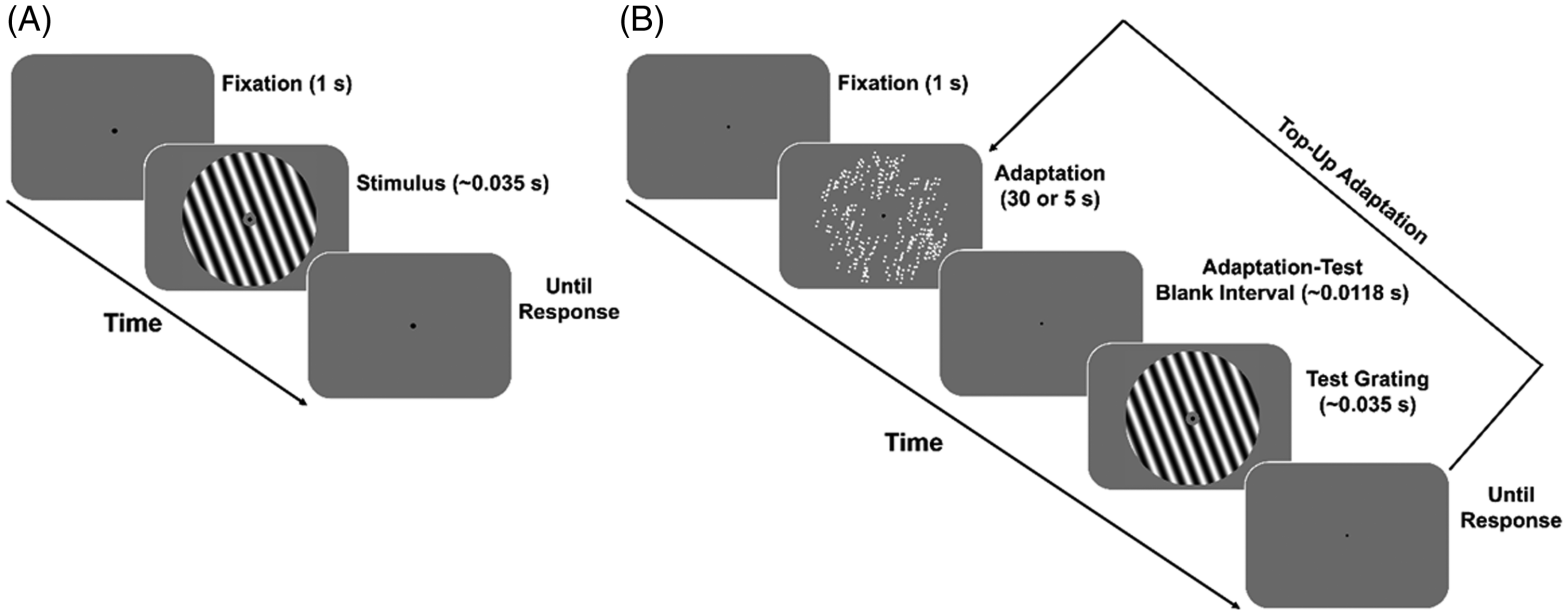
Representation of the procedure used in Experiment 1. (A) Baseline (no-adaptation) condition. (B) GP adaptation condition. For illustrative purposes, the adapting GP is represented with lower dipole density, wider dots, and interdot distance. The grating is represented with lower spatial frequency and higher contrast than the actual parameters used in Experiment 1.

Between conditions, participants were given a 5-minute rest in a lit room to avoid cumulative adaptation effects between trials, and a longer 10-minute break intervened after the first four conditions to avoid both fatigue and cumulative adaptation effects (Wolfe & O’Connell, 1986). As an additional precaution against cumulative adaptation effects, the sequence, in which adaptation to the different temporal frequencies was conducted, was randomized. The order in which the eight conditions were displayed was randomized across participants. Figure 2 shows the procedure used in Experiment 1.

The PSV estimated in the baseline condition was subtracted from the PSV estimated following adaptation to translational GPs; this removed the bias in observers’ orientation judgement that is common to the baseline and adapting conditions (Apthorp & Alais, 2009; Hawley & Keeble, 2006; Joung et al., 2000; Pavan et al., 2016). The resulting value was the magnitude of the TAE for each adapting temporal frequency and static adaptation.

The perceptual bias introduced by GP adaptation was estimated from the PSV, while the variation of accuracy in discriminating whether the grating was tilted clockwise or counterclockwise from vertical was derived from the slope of the psychometric function. The slope of the psychometric function therefore provides an estimate of test stimulus discriminability. The interest in the latter quantity stems from experimental evidence that adaptation might have an effect not only on the threshold but also on the slope of the psychometric function (Erlikhman et al., 2019; Morgan, 2014; Price & Prescott, 2012; Wright & Johnston, 1985). The slope values estimated from each staircase of the baseline blocks were averaged. The mean of the slopes for the no-adaptation baseline condition were also weighted by the SE values of the slope obtained from the curve fitting routine.

### Results

**Figure 3.**
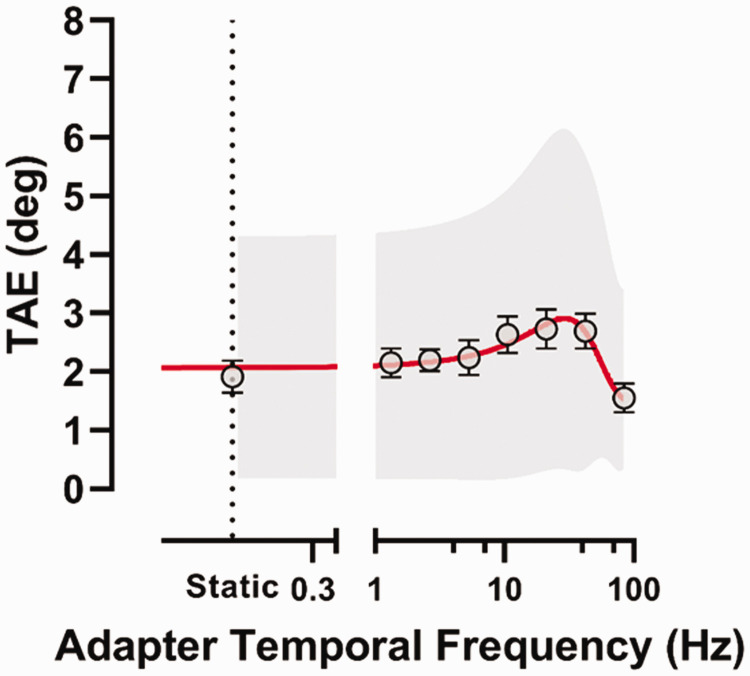
Tilt after-effect (deg) as a function of the adapting translational GP temporal frequency. The TAE for the static condition (0 Hz) is also reported. The abscissa is in *log* scale with a gap between 0.5 and 1.0 Hz. The red continuous line represents the Gaussian function fitted to the TAE values. The vertical dashed line indicates the TAE obtained in the static GP adaptation condition. Error bars ± SEM. The shaded grey area represents 95% confidence interval for the fitted Gaussian function. TAE = tilt after-effect.

Figure 3 shows the results of Experiment 1. A Shapiro–Wilk test for normality, conducted separately for the static condition and each temporal frequency of the adapting GP, reported that residuals were normally distributed (*p* > .05). A repeated-measures analysis of variance (ANOVA) revealed a significant effect of the adapting temporal frequency, *F*(7, 56) = 5.68, *p* = .0001, *partial η*^2^ = 0.42. Post hoc comparisons corrected with false discovery rate (FDR) at α = .05 (Benjamini & Hochberg, 1995) between the adapting temporal frequencies used are reported in Table 2. Post hoc comparisons revealed a significant difference between TAEs for static GPs and dynamic GPs at 10.59, 21.19, and 42.37 Hz (all adjusted *p* = .040), with dynamic GPs inducing stronger TAEs than the static adapting condition. In addition, the TAE estimated when adapting to the higher temporal frequency (i.e., 84.75 Hz) is lower than all the other adapting temporal frequencies but not when compared to the static condition and the 1.32 Hz adapting condition. It should also be noted that below 10.59 Hz there was not a significant difference with the static condition, suggesting that lower temporal frequencies did not induce stronger TAEs than the static adapting condition.

**Table 2.** Adjusted *p* Values for Multiple Post Hoc Comparisons Between the GP Temporal Frequencies Used in Experiment 1.

GP temporal frequency (Hz)	0 (Static)	1.32	2.65	5.30	10.59	21.19	42.37
0 (Static)							
1.32	.413						
2.65	.336	.87					
5.30	.255	.842	.87				
10.59	.023*	.101	.127	.177			
21.19	.007*	.062	.077	.1	.841		
42.37	.011*	.077	.1	.126	.87	.87	
84.75	.217	.053	.038*	.028*	<.001*	<.001*	<.001*

*Note*. The asterisks represent the significant comparisons. GP = Glass pattern.

We also performed eight FDR-corrected one-sample *t* tests between each adaptation condition and zero to test whether the TAE values were significantly higher than zero (i.e., no TAE). All the *t* tests resulted significant (all adjusted *p* < .001).

To determine the temporal frequency of the adapting GP at which the TAE peaked and to visualize the temporal frequency selectivity of neural populations responding to static and dynamic GPs, TAE values were fitted with both log-normal (Maniglia et al., 2015; Yu et al., 2010) and Gaussian functions. The best fitting function was a Gaussian function (see Supplementary Materials for equations and fitting results). The Gaussian fit to the TAE values is reported in Figure 3. The peak TAE was found for an adapter temporal frequency of 29.34 Hz, corresponding to a TAE of 2.91 deg.

**Figure 4.**
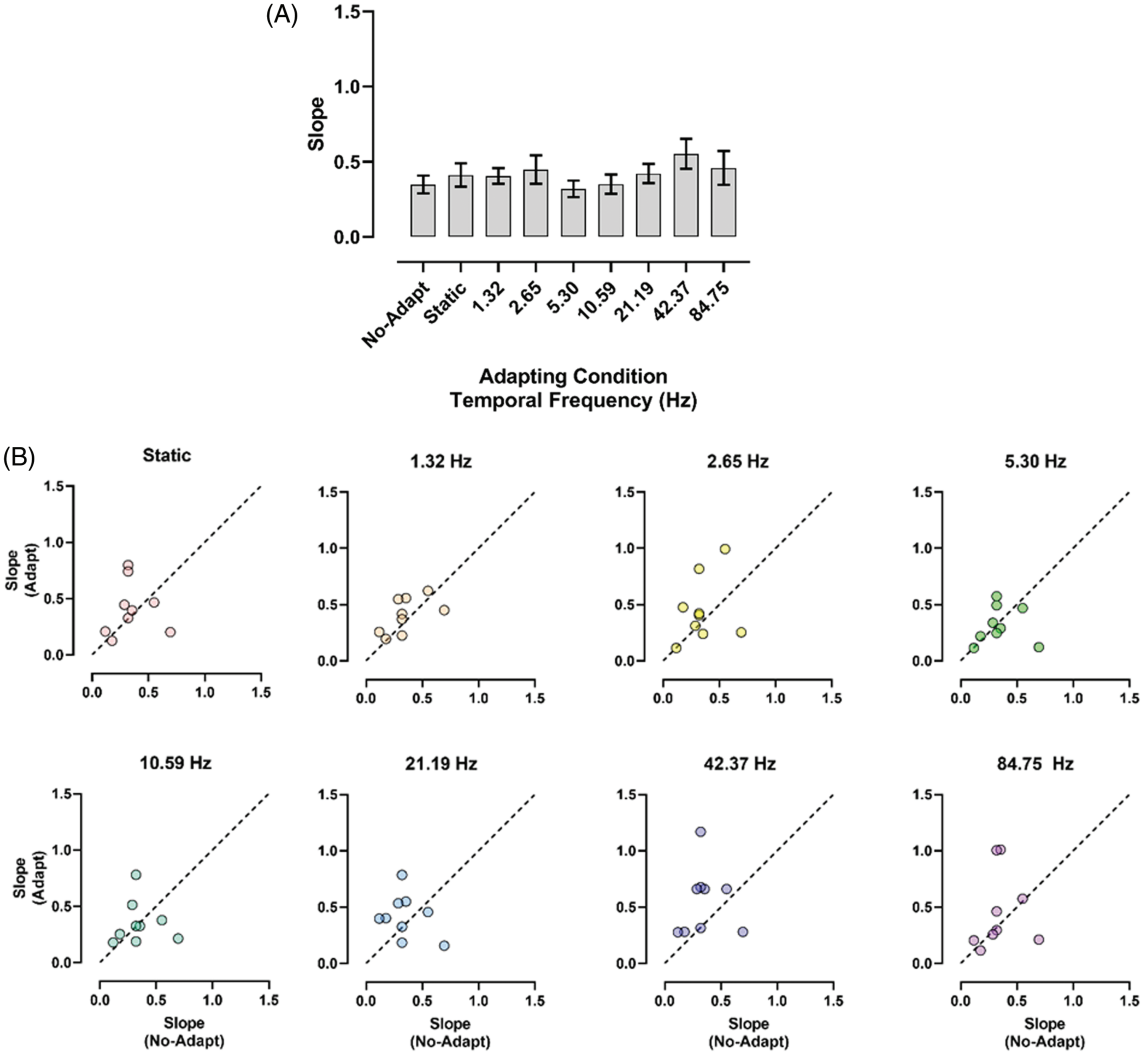
(A) Slopes estimated in Experiment 1 for the no-adaptation, static condition, and all the adapting GP temporal frequencies. Error bars ±SEM. (B) Individual slope values estimated in the adapting conditions as a function of slopes estimated in the no-adaptation condition. The diagonal dashed line indicates equal slopes for the adaptation and no-adaptation conditions.

Figure 4A shows the slopes estimated for the adapting conditions used. A repeated-measures ANOVA on slopes was conducted including as a within-subjects factor the adapting condition (i.e., no-adaptation, the static adaptation condition, and the seven temporal frequencies). The repeated-measures ANOVA showed no significant effect of the adaptation condition, *F*(8, 64) = 1.45, *p* = .19, *partial η*^2^ = 0.15. In Figure 4B, individual slope values estimated for the static and dynamic adaptation conditions are reported as a function of the slopes estimated in the no-adaptation condition. In general, points fall either on the diagonal line or are nearly equally scattered above and below the diagonal, indicating approximately the same slope values for each adapting condition.

### Discussion

In Experiment 1, we found that the temporal frequency manipulation produced quite noisy data (see, e.g., the 95% confidence band for the fitted Gaussian function in Figure 3), suggesting that TAE from adaptation to translational dynamic translational GPs is weakly tuned to the adapting temporal frequency, peaking at 29.34 Hz. Importantly, adaptation to static and dynamic translational GPs does not affect the discriminability of the test stimulus, with slopes being approximately the same between no-adaptation and adaptation conditions. Overall, these results suggest that dynamic GPs produce stronger orientation adaptation than static GPs, but the effect drops when using very high temporal frequencies, possibly because these temporal frequencies (e.g., 84.75 Hz) do not allow optimal temporal summation, thus affecting the quality of the orientation signal generated by the adapting pattern. In fact, it should be noted that temporal frequencies at 42.37 Hz and 84.75 Hz are beyond the temporal resolution of the human visual system (Robson, 1966). However, in these adapting conditions, the display was not perceived as a uniform white field, but observers could still perceive the orientation of the GPs given that the TAE in these conditions is significantly above zero. Besides, it should be noted that below 10 Hz, there was not a significant difference from the static condition, suggesting that there might be a specific range of temporal frequencies (i.e., between 10 Hz and 40 Hz) for optimal temporal integration in dynamic GPs. Previous studies using dynamic GPs found generally lower coherence detection thresholds for dynamic GPs than static GPs (Nankoo et al., 2015; Pavan et al., 2019). This suggests the involvement of a temporal summation mechanism in addition to spatial summation (Burr & Ross, 2006; Nankoo et al., 2015; Or et al., 2007; Pavan, Ghin, et al., 2017) and that temporal summation is optimal, in the sense that the TAE is maximized, when the interval between successive frames is between 24 ms and 94 ms, peaking at approximately 34 ms.

## Experiment 2

In Experiment 2, the adapting dipoles’ space constant (i.e., dipoles’ interdot distance) was varied, to test the dependence of the TAE on the relation between dipole space constant and test grating spatial frequency. The prediction was that for short and large dipoles’ interdot distance, the orientation content of the textures would be degraded (Dakin, 1997b), and consequently the TAE magnitude would be strongly reduced. The peak TAE was expected when the spatial content of the dynamic GP and the spatial frequency of the test grating matched.

### Method

#### Participants

One of the authors (“D. W. A.”) and a new sample of nine naïve participants took part in Experiment 2. All participants had normal or corrected to normal vision. Written informed consent was obtained from each participant prior to enrolment in the study.

#### Stimuli

**Figure 5.**
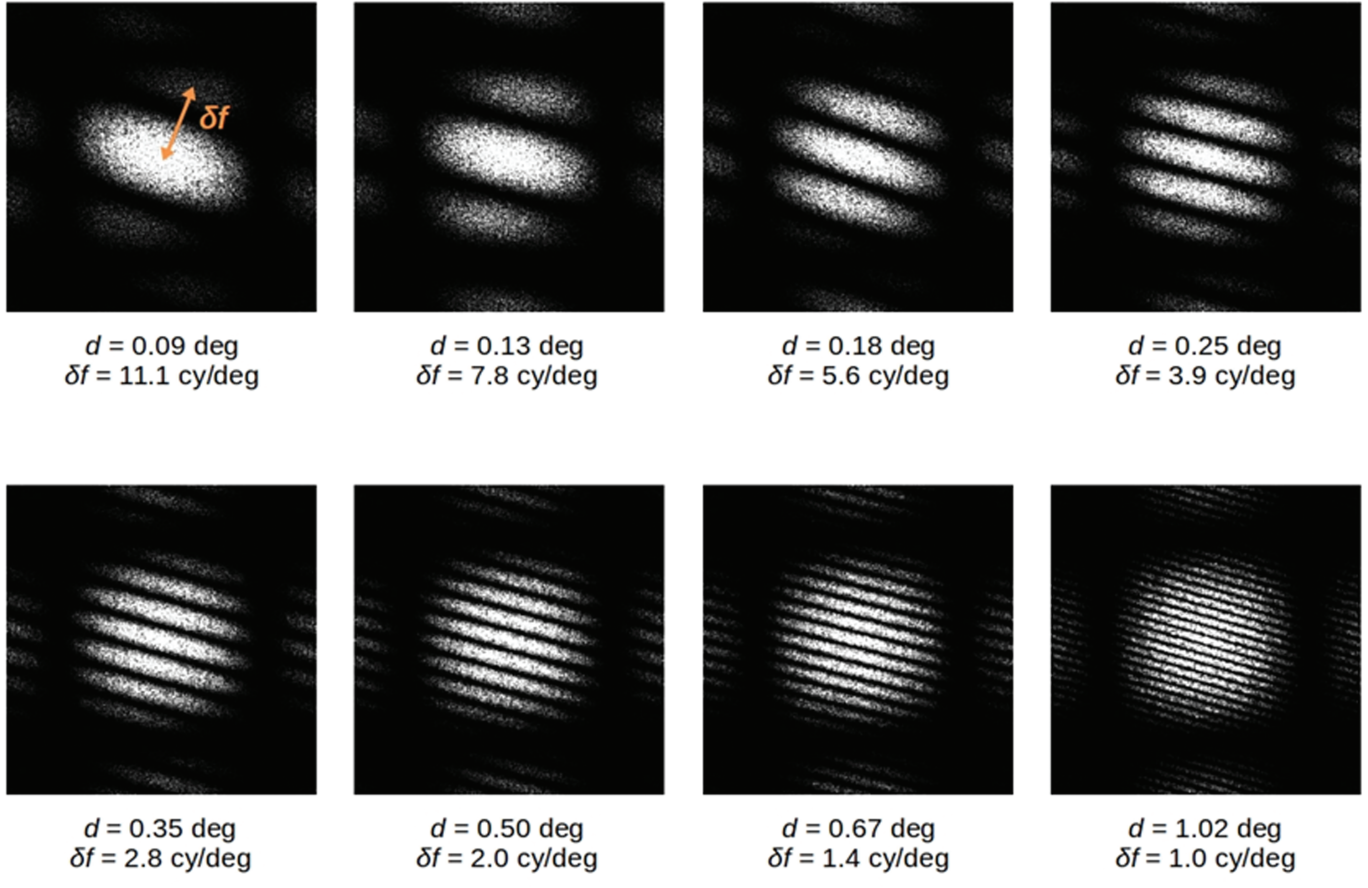
Frequency power spectra of eight GPs with different dipole distances (*d*) used in Experiment 2. Frequency power spectra were computed using the fast Fourier transform algorithm. *δf* are the frequency intervals between two consecutive bands and are inversely proportional to the dipole distances (Barlow & Olshausen, 2004; Burr & Ross, 2006). It is worth stressing that, because the images depict frequency spectra, the frequency intervals *δf* increase in the direction of *shorter* dipole distances (*d*).

Stimuli and procedure were the same as in Experiment 1. However, in Experiment 2, we used only a dynamic adapting GP with a temporal frequency of 21.19 Hz, because this was the closest temporal frequency to the peak frequency estimated in Experiment 1. Eight different dipoles’ interdot distances were used (Table 3). Figure 5 shows the frequency power spectra of eight GPs with varying dipole distances (*d*). The spectra have the appearance of a grating (orthogonally oriented to the GP orientation of 15°) overlapped to a Gaussian window centred in the origin of the frequency coordinates. In particular, the frequency intervals (*δf*) between two consecutive bands are inversely proportional to the dipole distances: direct measurements of such intervals (visually represented on the first spectrum of Figure 5) show that *δf =* 1/*d*.

**Table 3.** Dipoles’ Interdot Separation (deg) and Corresponding Spatial Frequency (c/deg) of the GP Calculated as the Reciprocal of the Interdot Distance.

*Interdot distance (deg)*							
0.09	0.13	0.18	0.25	0.35	0.5	0.67	1.02
*Corresponding spatial frequency (c/deg)*							
11.1	7.85	5.56	4.00	2.87	2.00	1.49	0.98

Observers performed two blocks of the no-adaptation (baseline) condition. For the baseline condition, two simple one-up/one-down staircases were randomly interleaved in each block, with one staircase starting with the grating tilted 10 deg clockwise and the other starting with the grating tilted 10 deg counterclockwise from vertical. The grating was rotated by 1 deg until the first reversal and then by 0.5 deg. Each staircase terminated after 75 trials. Observers performed one block of the adaptation condition for each interdot distance. In the adaptation condition, the staircase could start presenting the test grating tilted 8 deg either clockwise or counterclockwise from vertical, with the same step sizes as in the baseline condition. The adapting orientation was kept constant at 15 deg clockwise from vertical. The staircase terminated after 60 trials. The presentation order of no-adaptation and adaptation blocks was randomized across participants.

### Results

**Figure 6.**
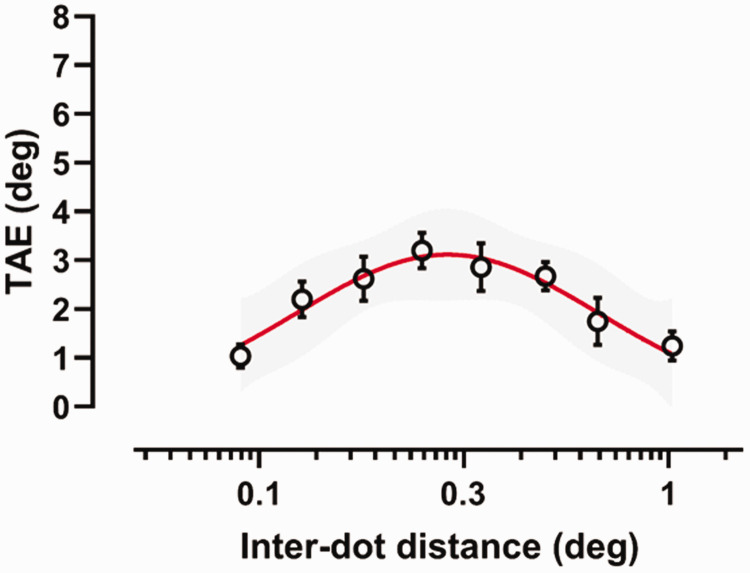
Tilt after-effect (deg) as a function of the dipoles’ interdot distance of the dynamic adapting GP. The abscissa is in *log* scale. The red continuous line represents the log-normal function fitted to the TAE values. Error bars ±SEM. The shaded grey area represents 95% confidence band. TAE = tilt after-effect.

Figure 6 shows the results of Experiment 2. A Shapiro–Wilk test for normality, conducted separately for each interdot distance of the adapting GP, reported that residuals were normally distributed (*p* > .05). A repeated-measures ANOVA revealed a significant effect of the interdot distance, *F*(7, 63) = 14.64, *p* = .0001, *partial η*^2^ = 0.62. FDR-corrected post hoc comparisons between the interdot distances are reported in Table 4. The results show that the shorter and the longer interdot distances (i.e., 0.09, 0.13 and 1.02 deg) elicited weaker TAEs than intermediate interdot distances.

**Table 4.** Adjusted *p* Values for Multiple Post Hoc Comparisons Between the Interdot Distances Used in Experiment 2.

Interdot distance (deg)	0.09	0.13	0.18	0.25	0.35	0.5	0.67
0.09							
0.13	.219						
0.18	<.0001*	.008*					
0.25	.0056	.0065*	.613				
0.35	<.0001*	<.0001*	.052	.255			
0.5	.0065*	.074	.894	.538	.11		
0.67	.0065*	.153	.123	.059	<.0001*	.219	
1.02	.486	.089	<.0001*	.0035*	<.0001*	.0056*	.0035*

*Note*. The asterisks represent significant comparisons.

We also performed eight FDR-corrected one-sample *t* tests between each adaptation condition and zero to test whether the TAE magnitude estimated for each adaptation condition was significantly higher than zero (i.e., no TAE). All the *t* tests resulted significant (all adjusted *p* ≤ 0.005). We also compared the TAEs estimated in Experiments 1 and 2 for the same condition: temporal frequency of the dynamic GP at 21.19 Hz and dipole distance of 0.18 deg. A paired sample *t* test revealed no significant difference between the two experiments for the same condition, *t*(17) = 0.12, *p* = .91, *Cohen’s d* = 0.055.

To determine the interdot distance of the adapting GP textures at which the TAE peaked, TAE values were fitted with the same log-normal and Gaussian functions as used in the first experiment. In Experiment 2, the best fitting function was the log-normal function. The peak TAE was found for an interdot distance of 0.29 deg, corresponding to a spatial frequency of 3.45 c/deg and producing a TAE magnitude of 3.12 deg.

**Figure 7.**
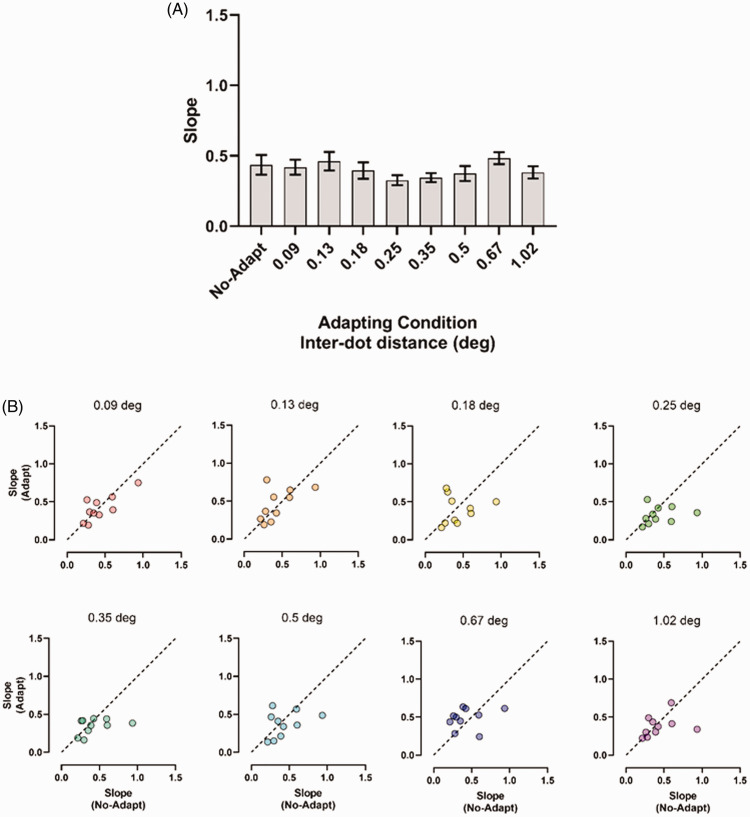
(A) Slopes estimated in Experiment 2 for the no-adapting condition and all the interdot distances used. Error bars ± SEM. (B) Individual slope values estimated in the adapting conditions as a function of slopes estimated in the no-adaptation condition. The diagonal dashed line indicates equal slopes for the adaptation and no-adaptation conditions.

Figure 7A shows the slopes estimated for each adapting condition. A repeated-measures ANOVA including as within-subjects factor the slopes estimated in the no-adaptation condition (baseline), in the static adaptation condition and the seven dynamic adaptation conditions with different interdot distances, did not report a significant effect of the adaptation condition, *F*(4.16, 37.43) = 1.28, *p* = .29, *partial η*^2^ = 0.12. Greenhouse–Geisser correction for degrees of freedom was used because the sphericity assumption was violated. In Figure 7B, individual slope values estimated for the adaptation conditions are reported as a function of the slopes estimated in the no-adaptation condition. As for Experiment 1, points fall either on the diagonal line or are nearly equally scattered above and below the diagonal, indicating approximately the same slope values for each adapting condition.

### Discussion

Experiment 2 locates the peak response in an intermediate range of interdot distances (0.29 deg). As reported in the Stimuli section, the frequency band intervals of a GP’s frequency spectrum are inversely proportional to the interdot distance. From a physiological point of view, it is well known that the visual system relies on the frequency domain transformation of the retinal image, which was initially represented through Fourier analysis (Graham, 1981). Although more refined, locally supported models of sparse image coding (e.g., Gabor- or wavelet-based analysis) have subsequently replaced the global Fourier approach, the concept of representing the neural image in the frequency domain remains valid, and the spectrum remains a meaningful indicator of the frequency composition of an image (see Gladilin & Eils, 2015 for details on an extension of the Fourier approach in vision). Therefore, the manipulation of dipoles’ spatial constant can be considered as a variation of the spatial frequency content of the adapting GP (see Figure 5). Accordingly, the results suggest that units combining motion and forming information exhibit selectivity for intermediate spatial frequencies (i.e., 3.45 c/deg). In general, this points again to a low- or intermediate level of motion-form integration for translational dynamic GPs. So far, our results show that form and motion integration in dynamic translational GPs relies on specific temporal and spatial properties of the stimulus.

## General Discussion

In this study, we investigated whether the spatiotemporal properties of adapting GPs and test gratings modulate the magnitude of the TAE. Participants were adapted to both static and dynamic translational GPs. After the adaptation period, they were required to judge the orientation of a briefly presented test grating.

The results from Experiment 1 show that the adapting temporal frequency does have a significant effect on the TAE magnitude. The findings showed that the greatest TAE was obtained by adaptation to dynamic translational GPs with a temporal frequency of approximately 30 Hz, corresponding to a time constant of approximately 34 ms, while the lowest TAE magnitude was produced by the static condition and with the temporal frequency at 84.75 Hz. The latter corresponds to an interframe interval equal to approximatively 12 ms. Such an interval might be too fast for producing a sizeable neuronal response so that the benefit of sampling of a higher quantity of dipoles across frames is counteracted by the modest activation produced by each frame. Because of different experimental paradigms and scarce evidence of the effects of the temporal frequency on dynamic GPs, there is not yet a consensus on how dynamic GPs presented at different temporal frequencies are processed along the visual pathway. Day and Palomares (2014), using circular GPs, investigated the effects of different temporal frequencies by presenting dynamic GP between 1.0 and 36 Hz. Their results showed a negative correlation between the temporal frequency of the pattern and the coherence threshold: The higher the temporal frequency, the lower the coherence threshold. Subsequently, Nankoo et al. (2015) investigated the effect of temporal frequency on translational dynamic GPs perception. The authors did not find a significant difference in detection thresholds when stimuli were composed of more than two unique GP frames and were presented between 20 and 60 Hz. Similarly, in our study, we found stronger TAE magnitude when the adapting GP temporal frequency was between 10.59 and 42.37 Hz, suggesting that there might be a specific range of temporal frequencies for optimal temporal summation, which is a process in which the neurons combine both the temporal and spatial information of a stimulus (Day & Palomares, 2014; Nankoo et al., 2012; Pavan, Ghin, et al., 2017). Importantly, our results also showed that increasing the adapting temporal frequency up to 84.75 Hz, the TAE magnitude was not different from the static adapting condition. These results support the notion that temporal summation plays an important role in the perception of global form from dynamic GPs, and it largely depends on the frequency at which the pattern is updated. It is plausible that global orientation from dynamic GPs is extracted at a level in which neurons combine motion and form information and are orientation-selective and broadly bandpass tuned to intermediate temporal frequencies (i.e., between 10 and 40 Hz). Moreover, our results indicate that dynamic GPs at the optimal temporal frequency are likely to provide the strongest orientation signals. In fact, the temporal updating of the GP may cause the perception of a larger number of dipoles within the physiological window of temporal integration. On the other hand, shorter frame durations may result in reduced perceived contrasts. These two concurring effects may aggregate into an optimal frame rate, synced to the temporal size of the integration window.

Concerning the possible neural correlates of translational GPs, the results of Experiment 1 point towards orientation-selective neural populations for translational GPs at intermediate levels of visual processing, possibly beyond V2. Indeed, previous cell recording studies on macaque monkeys showed that the mean optimum temporal frequency in V1 and V2 neurons for sinewave gratings of suprathreshold contrast drifting over the receptive field at the preferred orientation and direction is 4.4 and 4.7 Hz for V1 and V2, respectively (Foster et al., 1985). A similar temporal frequency tuning was observed in humans by Singh et al. (2000) using high contrast drifting gratings.

In addition, the TAE temporal frequency tuning found in Experiment 1 could depend on the temporal integration of dipoles with the extraction of the so-called motion streaks occurring from a low level of visual analysis up to human MT complex (hMT+) (Apthorp et al., 2013; Tang et al., 2015). *Motion streaks* or *speed lines* are trails of neural activity left behind fast-moving objects that can be detected by orientation-selective cells in the visual system. *Motion streaks* can influence the observer’s perception of motion direction by providing information about the axis of motion (Burr, 1980; Burr & Ross, 2002; Geisler, 1999; Ross, 2004; Ross et al., 2000). Apthorp et al. (2013), using fMRI, investigated the neural correlates of *motion streaks*. The authors measured the brain activity while participants observed either fast (inducing *motion streaks*) or slow drifting dots moving in different directions, or static-oriented stimuli. The authors found patterns of brain activity in early visual cortical areas distinguishing between different static orientations. A multivariate pattern classifier trained on the brain activity evoked by the static-oriented stimuli could then distinguish the direction of fast (*motion streaks*) but not slow motion. The authors found that the early visual cortex was activated for fast motion while hMT+ responded similarly to fast and slow motion. This indicates that directional fast motion that elicited *motion streaks* was not only processed by hMT+ but also by early visual areas (V1/V2). Pavan, Ghin, et al. (2017) used online rTMS to investigate the causal role of V1/V2 and hMT+ in the processing of dynamic (20 Hz) and static translational GPs. Participants were required to discriminate in which of two temporal intervals was presented a coherent and vertically oriented translational GP. The results showed that rTMS delivered over hMT+ impaired the perception of dynamic translational GPs, while rTMS delivered over V1/V2 impaired the perception of both static and dynamic translational GPs. The temporary disruption of V1/V2 could have impaired early form and motion integration preventing the extraction of *motion streaks*. Therefore, it is plausible that global orientation from dynamic translational GPs is extracted at a level in which neurons combine motion and form information, are orientation-selective, and broadly bandpass tuned to intermediate temporal frequencies (i.e., between 10 and 40 Hz).

Other studies investigated temporal integration mechanisms with different types of global structures (Gheorghiu & Erkelens, 2005; Hess & Ledgeway, 2003; Sharman et al., 2018). For example, Sharman et al. (2018) investigated the temporal characteristics and limits of symmetry perception. Stimuli were dynamic dot patterns consisting of either an ongoing alternation of two images (sustained presentation) or two images presented once (transient presentation) containing different amount of symmetry along the vertical axis. The authors varied the duration of the two images under different temporal conditions, from synchronous to delayed matched-pairs stimuli. The results showed that for the delayed conditions, sensitivity decreased gradually with longer image durations (>60 ms). In addition, spatial correlations across the symmetry midline could be integrated over time (∼120 ms), and symmetry mechanisms can tolerate temporal delays between symmetric dot-pairs of up to ∼60 ms. It should be noted that for dynamic GPs, we reported a similar integration window (from 24 ms up to ∼100 ms) peaking at 34 ms.

The spatial frequency selectivity also helps to understand how the visual system perceives specific visual stimuli (Henriksson et al., 2008; Kourtzi & Huberle, 2005; Kourtzi et al., 2003). In this regard, in Experiment 2, we manipulated the dipoles’ interdot distance that is the spatial frequency content of the dynamic translational GPs. The peak of TAE was found at a lower spatial constant of the GPs respect to the test pattern. These results support previous findings which show that orientation-selective low-level visual areas (e.g., V1/V2) are tuned to lower spatial frequencies than higher-level visual areas (e.g., V3; Altmann et al., 2003; De Valois et al., 1982; Foster et al., 1985; Hegdé & Van Essen, 2007; Henriksson et al., 2008; Kourtzi & Huberle, 2005; Kourtzi et al., 2003). The TAE greatest magnitude was produced by the lowest GP spatial frequencies, that is, those under the spatial constant of the adapting GP (5.56 c/deg—being the inverse of the interdot distance). As stated previously, this would suggest that neurons involved in low-level visual processing are orientation-selective and specifically selective to low spatial frequencies. The findings from Experiment 2 provided evidence that the low-level visual areas can process both *non-coherent motion* and form signals and also that units in these areas can combine these signals to form a global percept. In Experiment 2, the spatial period of the test grating was fixed at 0.18 deg, and the optimal dipole separation in terms of TAE magnitude was 0.29 deg, corresponding to a spatial frequency of 3.45 c/deg, that is approximately two times lower than the test grating spatial frequency used. Interestingly, De Valois et al. (1982) found that for macaque monkeys’ simple cells in the striate cortex, the spatial frequency peak is 3.0 c/deg, whereas for complex cells, it is 4.4 c/deg. This strongly suggests that adaptation to translational GPs may take place at the level of the striate cortex, up to visual area V2. Likewise, Smith et al. (2002, 2007) found that the best orientation selectivity for simple and complex V1 and V2 neurons occurred when dot separation in GPs was 0.25–0.5 of the optimal spatial period of the receptive field. This range corresponds to neurons with an optimal spatial frequency between 2.5 and 5.0 c/deg.

Our results can be explained considering a hierarchical model of the visual pathway (Wilson & Switkes, 2005; Wilson & Wilkinson, 1998; Wilson et al., 1997, 2004). However, feedback connectivity within the visual cortex can also play an important role (Angelucci et al., 2002; Lund et al., 2003). To this purpose, Roach et al. (2008), adapting to large complex global orientation structures (i.e., concentric), induced reliable remote TAE. The test stimuli were spatially limited Gabor patches. Their results showed that a certain degree of TAE was still present when adapter and test stimuli did not spatially overlap and had not the same spatial frequency. To explain their findings, the authors suggested that coding mechanisms for global form in extrastriate areas, activated during adaptation, would induce a feedback-driven inhibition of local orientation neurons in the early visual cortex, biasing the perceived orientation of the test Gabor patch. Accordingly, Liu et al. (2017), using magneto-encephalogram (MEG), provided further evidence on the role and the importance of recurrent connectivity in the processing of global form from dynamic GPs, showing that perceptual integration induced robust and rapid responses along the dorsal visual pathway in a reversed hierarchical manner. These results support an alternative model of global form processing, in which the dorsal visual pathway extracts very rapidly a coarse global form template, to subsequently guide low-level processing of visual information for its refinement.

In conclusion, our findings suggest that neural populations at low- and intermediate levels of visual analysis are tuned to a certain range of spatial and temporal frequencies and can combine form and *non-coherent motion*. Although psychophysical studies cannot provide specific information about downstream stages of analysis that integrate local information in GPs to provide signals about global form, the investigation of the spatiotemporal selectivity of orientation-selective neural populations from adaptation to translational GPs can provide clues about the processing stages.

## Supplemental Material

sj-pdf-1-ipe-10.1177_20416695211017924 - Supplemental material for Spatial and Temporal Selectivity of Translational Glass Patterns Assessed With the Tilt After-Effect Click here for additional data file. Supplemental material, sj-pdf-1-ipe-10.1177_20416695211017924 for Spatial and Temporal Selectivity of Translational Glass Patterns Assessed With the Tilt After-Effect by Andrea Pavan, Adriano Contillo, Filippo Ghin, Rita Donato, Matthew J. Foxwell, Daniel W. Atkins, George Mather and Gianluca Campana in i-Perception
