# Supplementary material for: Spatial and Temporal Selectivity of Translational Glass Patterns Assessed With the Tilt After-Effect
Source: Iperception. 2021 May 21;12(3):20416695211017924. doi: 10.1177/20416695211017924 (PMC8172339; doi:10.1177/20416695211017924)
Supplement: sj-pdf-1-ipe-10.1177_20416695211017924 - Supplemental material for Spatial and Temporal Selectivity of Translational Glass Patterns Assessed With the Tilt After-Effect [file sj-pdf-1-ipe-10.1177_20416695211017924.pdf]

Supplementary Material

Experiment 1

In order to determine the temporal frequency of the adapting GP at which the TAE peaked and to visualize the temporal frequency selectivity of neural populations responding to static and dynamic GPs, TAE values were fitted with both Log-Normal (Maniglia, Pavan, Aedo-Jury, & Trotter, 2015; Yu et al., 2010) and Gaussian functions. Both functions describe a trend with a local maximum surrounded by dampening tails. However, while the latter encodes a symmetry between the two tails, the former allows to incorporate asymmetries between them. The two functions are reported in Table S1. The Gaussian function is the same as that reported in Henriksson et al. (2008).

**Function name**	**Equation**	**Number of free parameters**
Log-Normal	$y = \frac{A}{x} e^{-\frac{(\ln(x)-\mu)^2}{2\sigma^2}} + B$	4
Gaussian	$y = Ae^{-\frac{(x-\mu)^2}{2\sigma^2}} + B$	4

Table S1. The two functions fitted to the TAE data. The functions have four parameters.

For both the Log-Normal and Gaussian functions, *y* is the TAE magnitude at a specific update rate, *x* are the adapting GP temporal frequencies (including the static condition, i.e., 0 Hz), *A* is the amplitude of the function, μ is the center (peak), σ is the standard deviation, and *B* is the offset of the function with respect to the ordinate. The two functions were fitted to the TAE values and then compared using the corrected AIC (*AICc*) index. It should be noted that the two functions have the same number of parameters and are not nested, therefore the comparison cannot be performed using an *F-test*. The estimated parameters are reported in Table S2. It should be noted that in the case of the Log-Normal function, the static GP corresponds to $\ln(0) = -\infty$. In order to avoid such divergence, the temporal frequency was set to a very small number, $10^{-4}\ Hz$. The robustness of such choice

was tested by varying the number from 10^{-6} to 10^{-2} Hz, which did not produce sensible variations in the fit outputs.

**Function**	**Parameters**	**Value**	**SE**	R^2	***AICc***
Log-Normal	*A*	20.41	0.326	0.64	-11.47
	μ	3.44			
	σ	0.75			
	B	1.97			
Gaussian	*A*	1.44	0.122	0.95	-27.14
	μ	29.34			
	σ	22.09			
	B	1.47			

Table S2. Parameters estimated for the log-Normal and Gaussian functions, including standard error (SE), R^2, and the *AICc* index. The delta between the *AICc* indexes is 15.67 (p = 0.0004). The likelihood that the Gaussian is the better model is 99.96%. For the Log-Normal function the peak is given by: $x_{peak} = e^{(\mu-\sigma^2)}$ and corresponds to 17.77 deg.

The Gaussian function had a lower *AICc* index and higher R^2 than the Log-Normal function, therefore the Gaussian function was selected as the best fitting model.

Experiment 2

In order to determine the inter-dot distance of the adapting GP textures at which the TAE peaked, TAE values were fitted with the same functions reported in Table S1. The estimated parameters are reported in Table S2.

Model	Parameters	Value	SE	R^2	*AICc*
Log-Normal	A	1.26	0.24	0.95	-16.56
	μ	-0.52			
	σ	0.85			
	B	0.073			
Gaussian	A	2.06	0.48	0.78	-5.15
	μ	0.36			
	σ	0.17			
	B	1.15			

Table S2. Parameters estimated for the log-Normal and Gaussian models fitted to the TAE values of Experiment 2. The delta between *AICc* indexes was 11.41 (p = 0.0033). The likelihood that the Log-Normal is the better model is 99.67%.

In this case, the Log-Normal fit had lower *AICc* index and higher R^2 than the Gaussian model. Therefore, the Log-Normal model was selected.
